# Effects of exercise on body fat percentage and cardiorespiratory fitness in sedentary adults: a systematic review and network meta-analysis

**DOI:** 10.3389/fpubh.2025.1624562

**Published:** 2025-07-17

**Authors:** Tian Huang, Huan Feng, Zhengwei Xie, Yubo Wang, Qingchuan Wang, Zhihua Wang

**Affiliations:** ^1^College of Physical Education, Sichuan University, Chengdu, China; ^2^College of Sports, Chengdu Sport University, Chengdu, China

**Keywords:** sedentary behavior, exercise intervention, body fat percentage, maximum oxygen uptake, peak oxygen uptake, network meta-analysis

## Abstract

**Background:**

Sedentary behavior is increasingly prevalent worldwide and associated with numerous health concerns including obesity and cardiovascular diseases. This study aimed to evaluate the comparative effectiveness of various exercise interventions on body fat percentage and cardiorespiratory fitness in sedentary adults.

**Methods:**

A systematic review and network meta-analysis of randomized controlled trials was conducted. Comprehensive searches were performed in PubMed, Embase, Web of Science, and Cochrane Library databases through December 10, 2024. All retrieved literature was imported into EndNote 21 for duplicate removal, and two reviewers independently screened articles and extracted data. Study quality was assessed using the ROB2 tool. Primary outcomes included body fat percentage (BF%), maximal oxygen uptake (VO₂max), and peak oxygen uptake (VO₂peak). Network meta-analysis used random-effects models with SUCRA ranking and low to moderate heterogeneity (I^2^ = 28–41%). Publication bias was assessed using funnel plots.

**Results:**

Fifty-one randomized controlled trials involving 2,201 participants were included. Risk of bias assessment showed 27 studies (52.9%) with low risk, 21 studies (41.2%) with some concerns, and 3 studies (5.9%) with high risk. Funnel plots indicated minimal publication bias. For BF% reduction, aerobic training ranked highest (SUCRA 97.5%), followed by resistance training combined with endurance training (SUCRA:78.2%) and aerobic training combined with strength training (SUCRA:77.4%). For VO₂max, strength training showed superior effectiveness (SUCRA:95.9%). For VO₂peak, aerobic training ranked highest (SUCRA:70.0%).

**Conclusion:**

This network meta-analysis demonstrates that aerobic training is most effective for reducing BF%, while strength training shows superior effectiveness for improving VO₂max in sedentary adults. Aerobic training also shows promise for enhancing VO₂peak. These findings provide evidence-based guidance for exercise prescription in sedentary populations, suggesting that different exercise modalities should be selected based on specific health improvement goals.

**Systematic Review Registration:**

PROSPERO (https://www.crd.york.ac.uk/prospero/display_record.php?RecordID=637089), identifier (CRD42025637089).

## 1 Introduction

With the rapid global socioeconomic development and technological advancement, sedentary behavior has become an undeniable health concern in modern lifestyle, carrying extensive and profound public health implications. Sedentary behavior is typically defined as stationary activities during waking hours with energy expenditure not exceeding 1.5 metabolic equivalents (METs), such as prolonged sitting for office work, watching television, using computers, or playing electronic games (1). Statistics indicate that globally, adults spend an average of over 4 h daily in sedentary behaviors, with even longer durations among certain occupational groups and in developing countries. This behavior is progressively replacing traditional physical activities, becoming an integral component of daily life (2). Research demonstrates that the widespread prevalence of sedentary behavior not only significantly increases obesity risk but is also closely associated with multiple health issues including metabolic disorders, cardiovascular diseases, certain cancers, and premature mortality (3, 4). Among these, body fat percentage (BF%), as a critical indicator of human health status, has a particularly close relationship with sedentary behavior. Sedentary behavior leads to gradual increases in body fat and induces obesity through mechanisms including reduced energy expenditure, suppression of fat metabolism-related enzyme activity, and increased insulin resistance (5). This phenomenon is especially prominent among adults with extended sedentary time, further exacerbating obesity-related health burdens (6). Additionally, the negative impact of prolonged sedentary behavior on cardiopulmonary function has gained increasing attention. M aximal oxygen uptake (VO₂max) and peak oxygen uptake (VO₂peak) are vital physiological indicators reflecting cardiorespiratory fitness. VO₂max represents the maximum rate of oxygen consumption during incremental exercise testing until exhaustion, while VO₂peak refers to the highest oxygen uptake achieved during exercise testing, particularly when true VO₂max criteria are not met (7), and their reduction often represents the primary manifestation of cardiopulmonary functional impairment due to sedentary behavior (8). Through multiple mechanisms including weakened myocardial pumping capacity, decreased muscle oxygenation efficiency, and inhibited mitochondrial metabolism, sedentary behavior causes significant decreases in maximum and VO₂peak, thereby increasing the risk of chronic diseases and reducing quality of life (9).

Exercise intervention, as an effective approach to mitigate health risks associated with sedentary behavior, has received increasing attention in recent years. By reducing sedentary time and increasing physical activity, exercise can not only lower BF% but also significantly improve Cardiorespiratory Fitness function, thereby alleviating health risks resulting from sedentary behavior (10). Aerobic training (AT, such as running, swimming, and cycling) is one of the most widely applied exercise intervention methods, which reduces body fat through increased energy expenditure while enhancing cardiopulmonary endurance and oxygen transport capacity, thus significantly improving VO₂max and VO₂peak (11). High-intensity interval training (HIIT), an emerging exercise intervention characterized by alternating high-intensity exercise with low-intensity intervals in short durations, not only offers time-efficiency advantages but also demonstrates great potential in improving VO₂max (12). Additionally, resistance training (RT) and low-intensity steady-state training (LISS) are widely applied in health interventions across different populations. RT primarily reduces BF% by increasing muscle strength and basal metabolic rate, while LISS achieves continuous energy expenditure through prolonged low-intensity exercise; however, its improvement on Cardiorespiratory Fitness function may not be as significant as HIIT (13). Despite extensive research on exercise interventions, significant knowledge gaps remain. Previous systematic reviews have primarily focused on pairwise comparisons between two exercise modalities, limiting the ability to simultaneously rank multiple interventions (14). No comprehensive network meta-analysis has specifically examined the relative effectiveness of different exercise types in sedentary adults—a population representing over 60% of adults globally (15) and contributing to 3.2 million preventable deaths annually (16). This evidence gap leaves clinicians without clear guidance for optimal exercise prescription, necessitating a network meta-analysis approach that can synthesize both direct and indirect evidence to provide hierarchical rankings of intervention effectiveness. Therefore, this study aims to systematically review and conduct a network meta-analysis to integrate existing evidence on the effects of exercise interventions on BF% and cardiorespiratory fitness (VO₂max and VO₂peak) in sedentary adults, providing evidence-based rankings to guide clinical practice and public health policy.

## 2 Materials and methods

This systematic review and meta-analysis was conducted in accordance with the Preferred Reporting Items for Systematic Reviews and Meta-Analyses (PRISMA) 2020 ^[17]^guidelines. The protocol was prospectively registered in the International Prospective Register of Systematic Reviews (PROSPERO) database (Registration ID: CRD42025637089).

### 2.1 Inclusion and exclusion criteria

#### 2.1.1 Study types

The included study type was randomized controlled trials (RCTs).

#### 2.1.2 Study participants the study participants were sedentary adults

The study participants were sedentary adults aged 18–65 years. Sedentary behavior was operationally defined as meeting any of the following criteria: (1) IPAQ total score <600 MET-minutes/week or classified as ‘low’ physical activity level; (2) Self-reported daily sitting time ≥6 h; (3) Meeting study-specific sedentary criteria as defined by original research with clear operational definitions.

#### 2.1.3 Interventions

The intervention groups received exercise interventions lasting no less than 5 weeks. The types of exercise interventions primarily included the following categories: AT; HIIT; moderate-intensity continuous training (MICT); RT, etc. The control groups consisted of any of the following: blank control (no exercise intervention); health education; or physical activities different from the intervention group’s exercise form.

#### 2.1.4 Outcome measures

At least one of the following outcome measures was assessed before and after intervention: BF%, VO₂max, VO₂peak.

#### 2.1.5 Inclusion and exclusion criteria

Inclusion and exclusion criteria is shown in Table 1.

**Table 1:** Inclusion and exclusion criteria.

Category	Specific criteria
Inclusion criteria	RCTs, limited to Chinese and English languages. Sedentary adults, with sedentary behavior defined as self-reported or assessed by physical activity questionnaires, with average daily sedentary time ≥6 h, or not engaging in regular physical activity (moderate-intensity activity <120 min per week). Control groups consisting of blank control (no intervention), health education, or exercise modalities different from the intervention group. Assessment of at least one primary outcome measure before and after intervention: BF%, VO₂max, VO₂peak.
Exclusion criteria	Patients with diabetes. Theses, conference abstracts, registration protocols, animal experimental studies, and literature for which full texts were unavailable. Studies from which valid data could not be extracted, and attempts to contact the authors were unsuccessful. Duplicate literature content or repeatedly published research data. Studies where participants were not defined as sedentary adults, or where sedentary behavior was not clearly reported.

### 2.2 literature search strategy

A search was conducted in Embase, Cochrane Library, Web of Science, and PubMed databases to collect RCTs on the effects of exercise interventions on body fat mass, VO₂max, and VO₂peak in sedentary adult populations. The search period extended from the establishment of each database to December 10, 2024, with language restriction limited to English. The search strategy employed Boolean logic, combining subject headings and free terms to enhance search comprehensiveness. Additionally, references of included studies were traced to exhaust all relevant research. Search terms included: Exercise, Sports, Physical, Athletic, Practice, Train, Sedentary, Sedentary behavior, Physical inactivity, Lack of physical activity, Randomized controlled trials as topic, Random, Clinic, Control, Trial, Adult, Aged, Adult, Older adult, Geriatric, Senior, etc. Searches included published studies only, with no attempt to identify unpublished studies or grey literature.

### 2.3 Literature screening and data extraction

Two researchers independently screened the literature and extracted data, following a systematic approach to ensure methodological rigor. All retrieved literature was imported into EndNote 21 (Clarivate Analytics, Philadelphia, PA, USA) for duplicate removal and preliminary organization. Initially, titles and abstracts were reviewed to exclude obviously irrelevant studies, followed by full-text assessment for final inclusion determination. For inaccessible full texts, corresponding authors were contacted via email, with articles excluded if no response was received within two weeks. All disagreements during the screening and extraction process were resolved through discussion or consultation with a third party. When information was missing from publications, efforts were made to contact original authors for supplementary data. The extracted data was organized in an Excel spreadsheet (version 16.0) with the following fields: author, publication year, country of origin, BMI (intervention/control groups), sedentary diagnostic criteria, intervention duration, number of participants in intervention group, number of participants in control group, intervention method, control condition, and outcomes. This structured approach enabled comprehensive analysis while maintaining data integrity throughout the review process.

### 2.4 Assessment of risk of Bias in included studies

Two researchers assessed the risk of bias in included studies using the ROB2 tool for RCTs as recommended by the Cochrane Handbook (17). The ROB2 tool encompasses five core domains: randomization process, evaluating the randomness and concealment of allocation sequences; bias due to deviations from intended interventions, assessing deviations in intervention implementation and blinding procedures; bias due to missing outcome data, evaluating the extent and handling methods of missing data; bias in measurement of the outcome, assessing consistency and reliability of outcome measurements; and bias in selection of the reported result, evaluating selective reporting (18, 19). Based on the risk of bias in each domain, studies were classified as “low risk,” “some concerns,” or “high risk.” Any disagreements during the evaluation process were resolved through discussion or consultation with a third party.

### 2.5 Statistical analysis

This study was based on a frequentist framework and utilized Stata 18 (StataCorp, College Station, TX, USA) and the “netmeta” package in R software (R Foundation for Statistical Computing, Vienna, Austria) for network meta-analysis. All effect sizes were reported as mean differences (MD) with 95% confidence intervals (CIs) to evaluate the reliability of estimates. All effect size pooling was based on random-effects models to fully account for potential heterogeneity between studies. Heterogeneity was assessed using I^2^statistics, with I^2^ > 50% indicating substantial heterogeneity. To comprehensively display comparisons between interventions, network evidence plots were constructed to visualize direct and indirect comparisons among different interventions. In these plots, nodes represent interventions with sizes proportional to intervention group sample sizes; connecting lines between nodes indicate direct comparison relationships, with line thickness proportional to the number of studies. The relative effectiveness of each intervention was assessed using surface under the cumulative ranking (SUCRA) curves. SUCRA values range from 0–1, with 1 indicating the most effective intervention and 0 indicating the least effective (20). To further analyze relative effects between interventions, this study employed league tables presenting pairwise comparisons between all interventions. League tables contain effect sizes (MD) and their 95% CIs between interventions, providing intuitive superiority comparisons (21). For consistency of network evidence, consistency tests were conducted, calculating consistency factors and their 95% CIs to evaluate consistency between direct and indirect evidence. If *p* > 0.05, the network model demonstrated consistency and a consistency model was used for analysis; if *p* ≤ 0.05, inconsistency existed and an inconsistency model was applied (22). Additionally, funnel plots were used to detect potential publication bias or small-study effects. Funnel plots reflect potential bias through the symmetry of effect size distribution; obvious asymmetry may suggest publication bias. All statistical analyses in this study were based on two-sided tests with significance level set at *p* < 0.05.

## 3 Results

### 3.1 Literature search results

**Figure 1:**
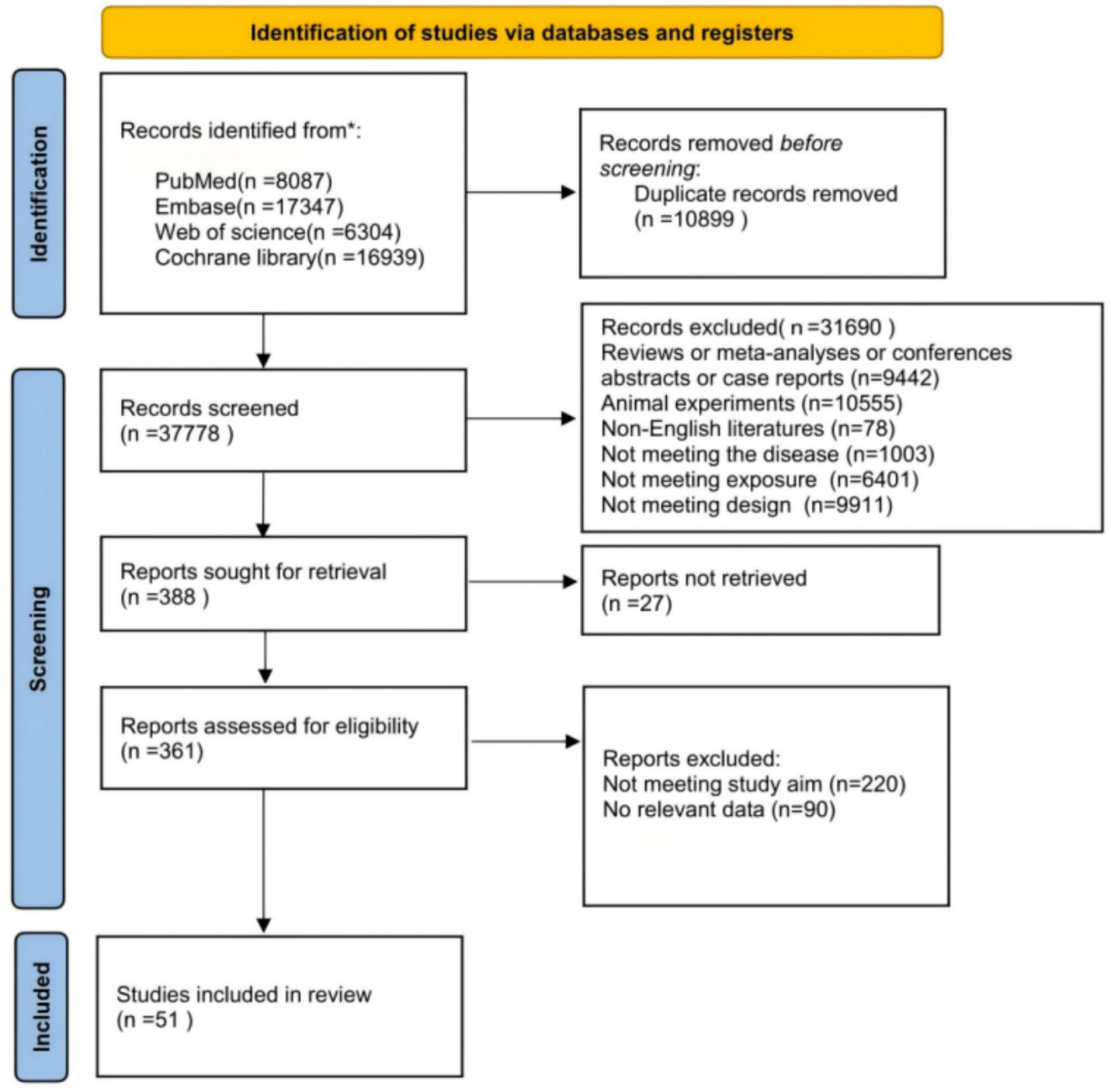
PRISMA flow diagram.

A total of 48,677 articles were retrieved through searches in PubMed, Embase, Web of Science, and Cochrane Library databases. After removing 10,899 duplicates, 37,778 articles proceeded to initial screening. Based on titles and abstracts, 31,690 articles were excluded as they did not meet the research objectives, including reviews, animal experiments, non-English literature, or articles that did not meet disease, exposure, or design criteria. The remaining 388 articles proceeded to full-text assessment, with 27 excluded due to inaccessible full texts. After evaluating 361 full-text articles against inclusion and exclusion criteria, 310 were eliminated. The primary reasons for exclusion included: studies not meeting the research objectives (*n* = 220) and studies with no relevant data (*n* = 90). Ultimately, 51 randomized controlled trials were included for analysis. The specific screening process is shown in Figure 1.

### 3.2 Basic characteristics of included studies

The 51 included randomized controlled trials involved 1,232 participants in intervention groups and 969 in control groups, with subjects aged 20–79 years and mean BMI ranges of 18.7–40.0 kg/m^2^. Sedentary behavior was defined primarily through International Physical Activity Questionnaire or self-reported assessments. Intervention durations ranged from 6 weeks to 24 months, with frequencies of 2–5 sessions per week.

Studies were predominantly conducted in the United States (*n* = 18, 35.3%), China (*n* = 5, 9.8%), United Kingdom (*n* = 4, 7.8%), with additional studies from Brazil, Spain, and other countries. Primary interventions included AT (*n* = 14), HIIT (*n* = 13), AT+RT (*n* = 11), MICT (*n* = 7), RT (*n* = 6), and various other exercise combinations. Study outcomes encompassed BF%, VO₂max, and VO₂peak. Detailed study characteristics are presented in Supplementary Table 1.

### 3.3 Risk of bias assessment results for included studies

**Figure 2:**
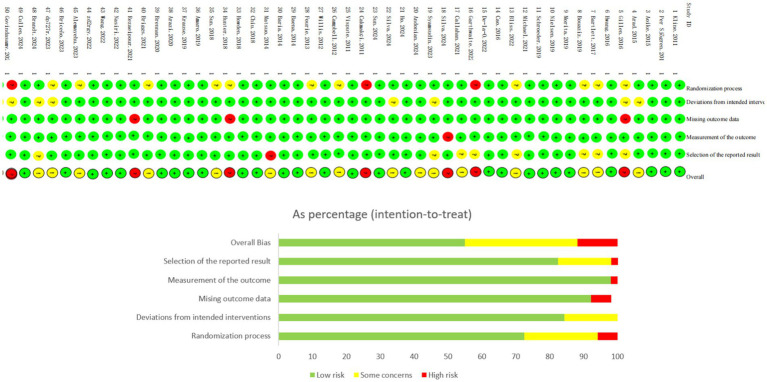
Risk of bias assessment results for included studies.

This study systematically evaluated 51 included studies using the Cochrane risk of bias assessment tool (ROB2). Regarding the “randomization process” domain, 35 studies were assessed as low risk of bias, while 16 studies had some concerns; in the “deviations from intended interventions” domain, 12 studies showed some concerns; for the “missing outcome data” domain, 11 studies presented some concerns; in the “measurement of the outcome” domain, 8 studies were evaluated as high risk of bias; and for the “selection of the reported result” domain, 9 studies exhibited selective reporting bias. Overall, 27 studies were assessed as low risk, 21 studies showed some concerns, and 3 studies were classified as high risk. In conclusion, the majority of included studies demonstrated high levels of bias control, with only a few studies showing high risk of bias. Detailed results are presented in Figure 2.

### 3.4 Network meta-analysis results

This study employed node-splitting method, loop inconsistency tests, and inconsistency models to examine network inconsistency for body fat mass, VO₂max, and VO₂peak. Loop inconsistency test results showed no significant inconsistency in all triangular loops, indicating good consistency between direct and indirect evidence. Inconsistency model testing further confirmed the consistency of outcome indicator analysis results (*p* > 0.05). Node-splitting method results revealed no statistically significant differences between direct and indirect comparisons for each outcome indicator (*p* > 0.05). Heterogeneity assessment showed low to moderate between-study heterogeneity across all outcome measures, with I^2^ values consistently below 50% for body fat percentage (I^2^ = 32%), VO₂max (I^2^ = 28%), and VO₂peak (I^2^ = 41%), indicating minimal heterogeneity and supporting the robustness of pooled estimates. Based on these test results, this study used a consistency model for analysis, yielding results with high stability and credibility (Supplementary Table 6).

#### 3.4.1 Body fat percentage

**Figure 3:**
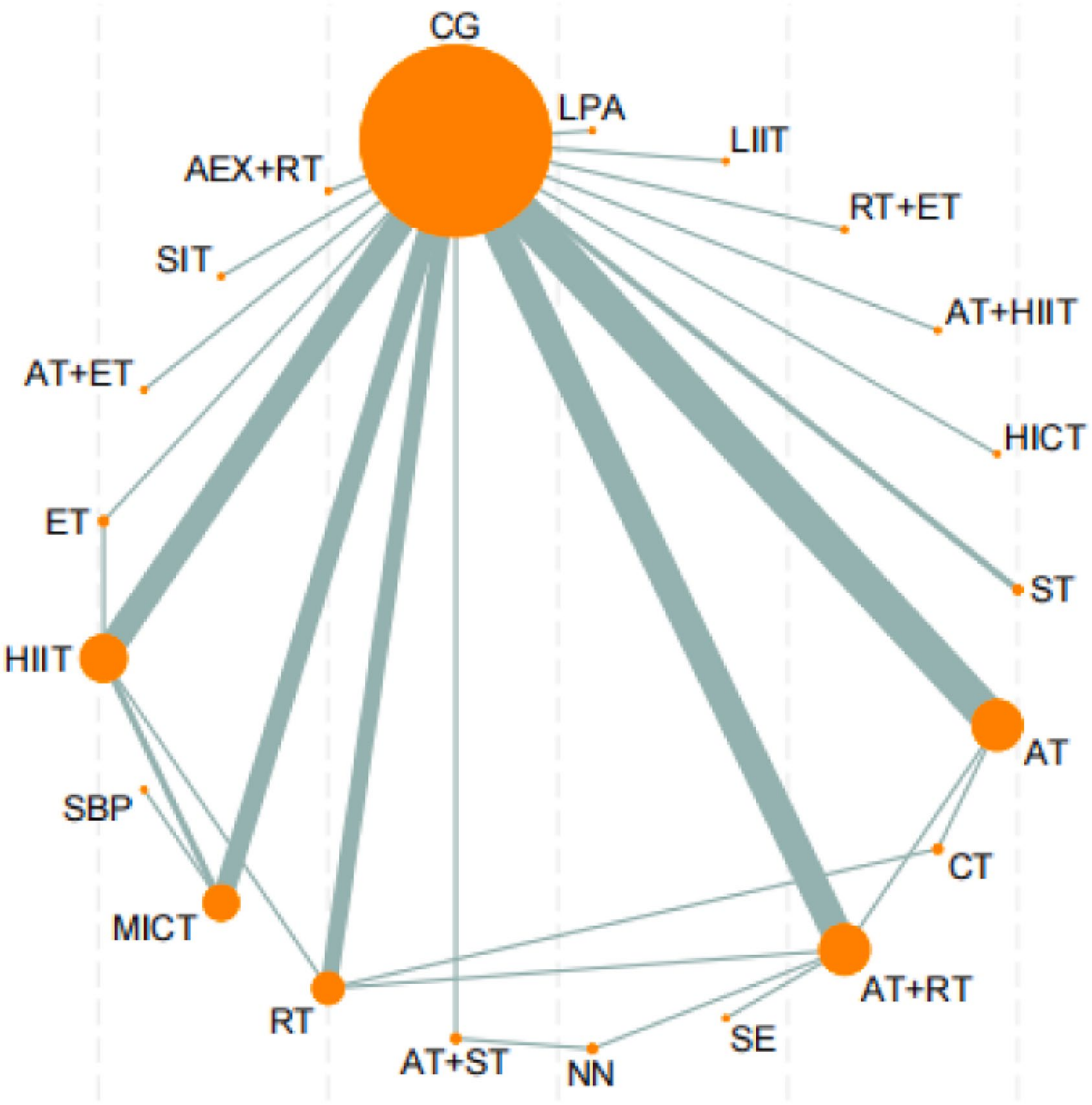
Network evidence diagram of the effects of different exercise. AT, aerobic training; RT, resistance training; ST, strength training; ET, endurance training; SE, stretching exercise; MICT, moderate-intensity continuous training; SIT, sprint interval training; MICT, moderate-intensity interval training; HICT, high-intensity circuit training; CG, control group; AEX, high-intensity continuous exercise; SBP, strengthened exercise program; NN, routine care; LIIT, low-intensity interval training; LPA, short-duration moderate-intensity physical activity.

The network meta-analysis results of 21 intervention measures (including control groups) for reducing BF% in sedentary adults (Figure 3) revealed that AT was significantly superior to ST (MD = 6.72, 95%CI: 0.82, 12.62, *p* < 0.05), SBP (MD = 9.14, 95%CI: 2.77, 15.52, *p* < 0.05), NN (MD = 8.17, 95%CI: 1.81, 14.53, *p* < 0.05), MICT (MD = 8.44, 95%CI: 2.69, 14.20, *p* < 0.05), LPA (MD = 8.68, 95%CI: 1.96, 15.40, *p* < 0.05), LIIT (MD = 8.78, 95%CI: 0.32, 17.24, *p* < 0.05), HIIT (MD = 7.92, 95%CI: 2.18, 13.66, *p* < 0.05), HICT (MD = 7.94, 95%CI: 0.10, 15.78, *p* < 0.05), ET (MD = 7.81, 95%CI: 1.22, 14.40, *p* < 0.05), CT (MD = 9.05, 95%CI: 2.36, 15.74, *p* < 0.05), CG (MD = 7.03, 95%CI: 0.99, 13.08, *p* < 0.05), AT+ST (MD = 6.00, 95%CI: 0.36, 11.65, *p* < 0.05), AT+RT (MD = 7.58, 95%CI: 1.37, 13.79, *p* < 0.05), AT+HIIT (MD = 9.08, 95%CI: 2.05, 16.11, *p* < 0.05), and AT+ET (MD = 6.99, 95%CI: 1.36, 12.63, *p* < 0.05).

Additionally, AT+ST demonstrated significantly superior effects compared to RT (MD = 1.43, 95%CI: 0.04, 2.82, *p* < 0.05), MICT (MD = 2.44, 95%CI: 0.53, 4.35, *p* < 0.05), and HIIT (MD = 1.91, 95%CI: 0.06, 3.76, *p* < 0.05). Furthermore, RT (MD = -1.24, 95%CI: −2.32, −0.16, *p* < 0.05), AT+ST (MD = -2.68, 95%CI: −3.77, −1.58, *p* < 0.05), AT+ET (MD = -1.69, 95%CI: −2.74, −0.63, *p* < 0.05), and AT (MD = -8.68, 95%CI: −14.22, −3.14, *p* < 0.05) all demonstrated significantly superior effects compared to AEX + AT. No significant differences were observed in other pairwise comparisons (Supplementary Table 3).

**Figure 4:**
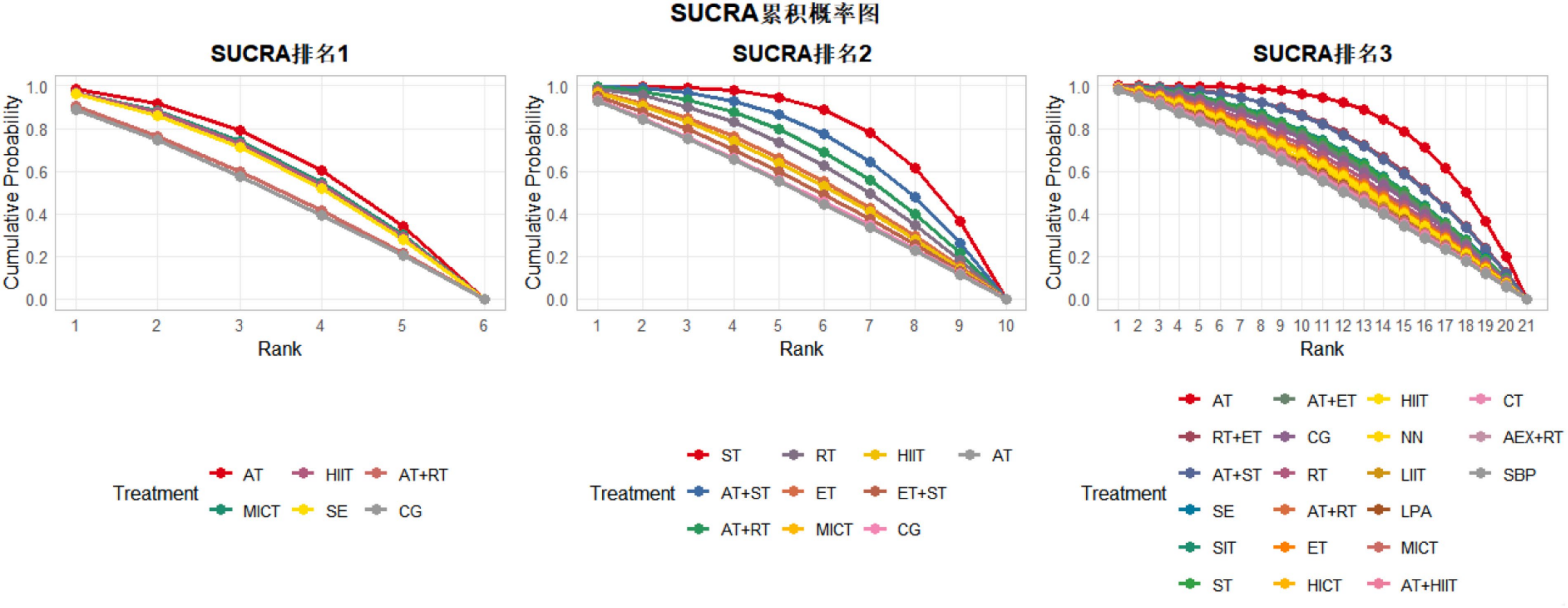
Cumulative probability ranking diagram of various exercise interventions on VO₂peak, VO₂max, and body fat percentage. SUCRA ranking 1, VO₂peak; SUCRA ranking 2, VO₂max; SUCRA ranking 3, body fat percentage; AT, aerobic training; RT, resistance training; ST, strength training; ET, endurance training; SE, stretching exercise; MICT, moderate-intensity continuous training; SIT, sprint interval training; MIIT, moderate-intensity interval training; HICT, high-intensity circuit training; CG, control group; AEX, high-intensity continuous exercise; SBP, strengthened exercise program; NN, routine care; LIIT, low-intensity interval training; LPA, short-duration moderate-intensity physical activity; FP, body fat percentage; VM, VO₂max; VP, VO₂peak.

**Figure 5:**
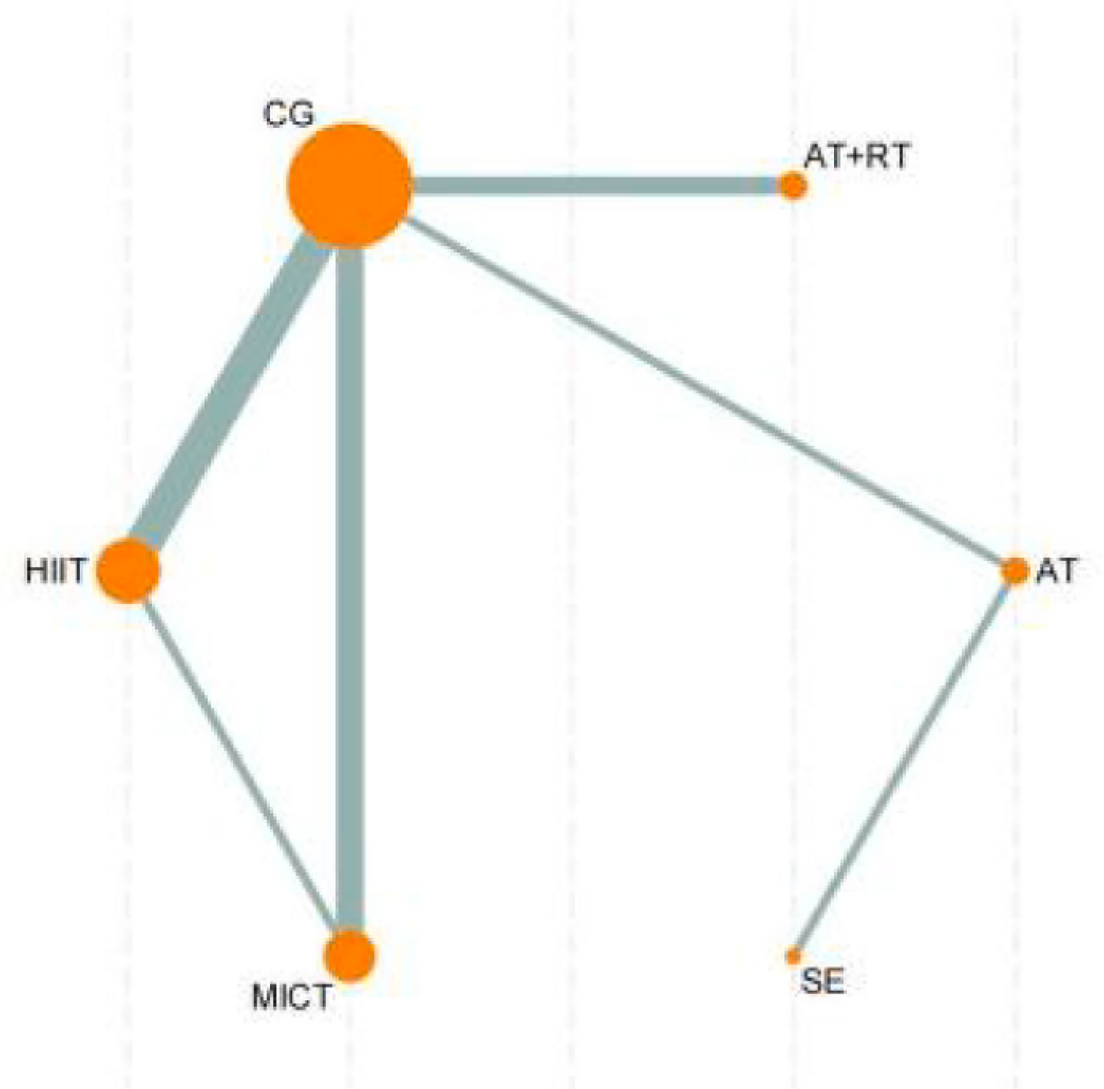
Network evidence diagram of the effects of different exercise modalities on VO₂peak in sedentary adults. CG, control group; HIIT, high-intensity interval training; MICT, moderate-intensity continuous training; SE, stretching exercise; AT, aerobic training; RT, resistance training.

According to the SUCRA ranking of all 21 intervention measures (including control groups), the top five interventions were: AT (97.5%), RT + ET (78.2%), AT+ST (77.4%), SIT (65.4%), and SE (65%). Rankings of other intervention measures are presented in Figure 4. For detailed information regarding all pairwise comparisons of interventions, please refer to Figures 4, 5.

#### 3.4.2 Cardiorespiratory fitness

##### 3.4.2.1 VO₂max

**Figure 6:**
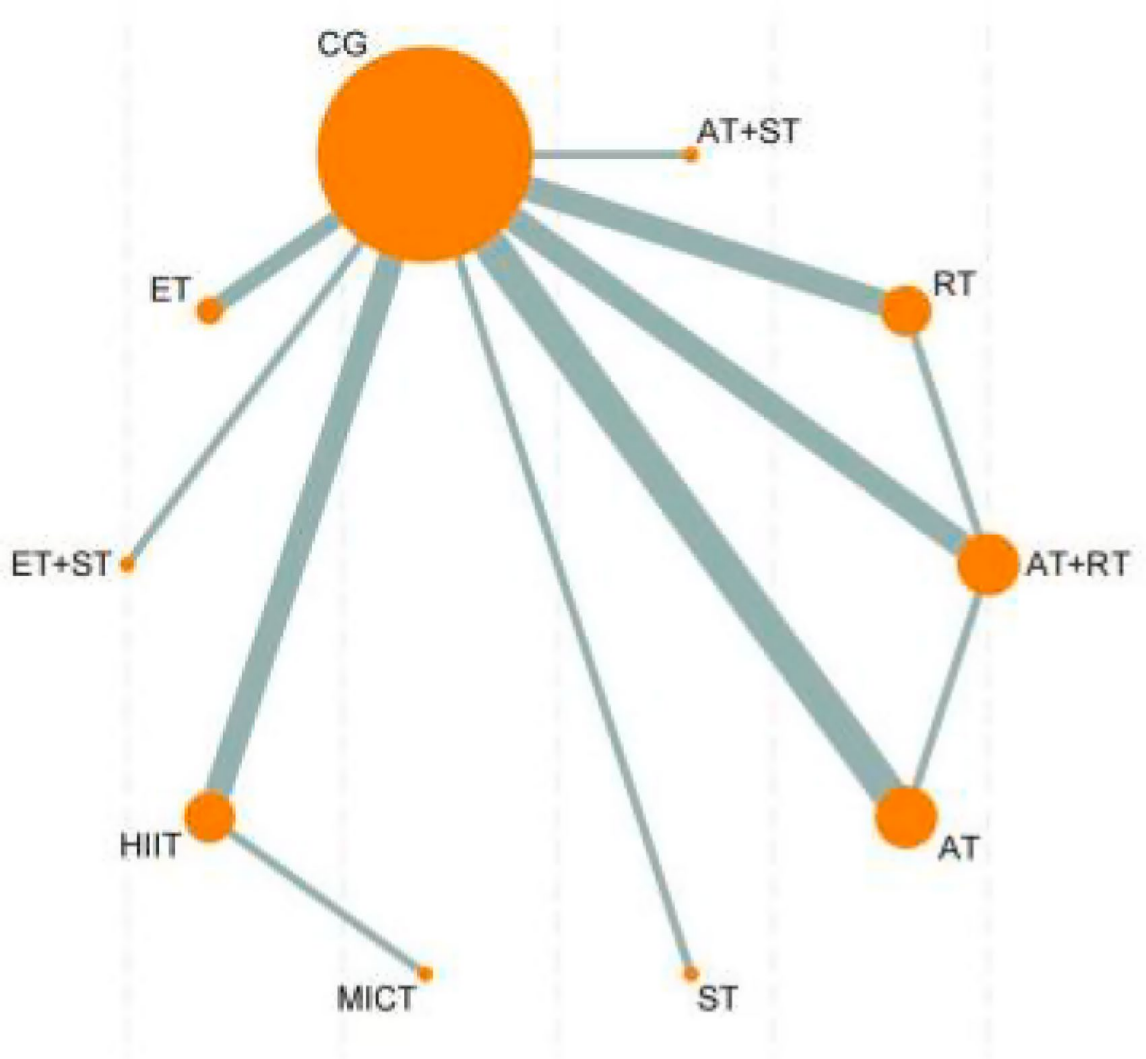
Network evidence diagram of the effects of different exercise. CG, control group; ET, endurance training; ST, strength training; HIIT, high-intensity interval training; MICT, moderate-intensity continuous training; AT, aerobic training; RT, resistance training.

The network meta-analysis results of 10 intervention measures (including control groups) for improving VO₂max in sedentary adults (Figure 6) showed that ST was significantly superior to CG (MD = 4.92, 95%CI: −8.39, −1.45, *p* < 0.05), ST + AT (MD = -6.02, 95%CI: −9.15, −2.89, *p* < 0.05), RT + AT (MD = -7.37, 95%CI: −10.77, −3.96, *p* < 0.05), and AT (MD = -11.29, 95%CI: −17.58, −5.00, *p* < 0.05). RT was significantly superior to RT + AT (MD = -7.67, 95%CI: −15.06, −0.27, *p* < 0.05) and AT (MD = -11.59, 95%CI: −20.68, −2.50, *p* < 0.05). Furthermore, MIIT (MD = -8.78, 95%CI: −16.75, −0.82, *p* < 0.05), HIIT (MD = -10.07, 95%CI: −19.56, −0.58, *p* < 0.05), and ET (MD = -8.10, 95%CI: −16.08, −0.12, *p* < 0.05) were all significantly superior to AT for improving VO₂max in sedentary adults. No significant differences were observed in other pairwise comparisons (Supplementary Table 4).

According to the SUCRA ranking of all 10 intervention measures (including control groups), the top five interventions were: ST (95.9%), ST + AT (81.8%), RT + AT (70.5%), RT (60.0%), and ET (46.0%). Rankings of other intervention measures are presented in Figure 4. For detailed information regarding all pairwise comparisons of interventions, please refer to Figure 4.

##### 3.4.2.2 VO₂peak

The network meta-analysis results of 6 intervention measures (including control groups) for improving VO₂peak in sedentary adults (Supplementary Table 5) showed no significant differences in pairwise comparisons. For detailed information, please refer to Appendix 1. According to the SUCRA ranking of all 6 intervention measures (including control groups), the results were: AT (70.0%) > MICT (61.0%) > HIIT (58.9%) > SE (55.5%) > RT + AT (30.4%) > CG (24.1%). For detailed information regarding all pairwise comparisons of interventions, please refer to Figure 4.

#### 3.4.3 Publication bias or small study effect testing

**Figure 7:**
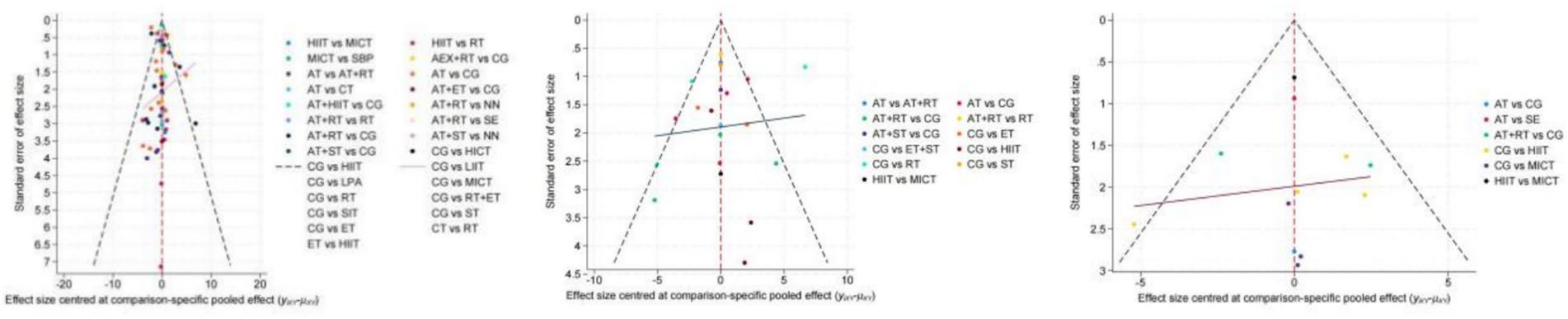
Funnel plots of various exercise interventions on body fat percentage, VO₂max, and VO₂peak.

Funnel plots were used to test for publication bias or small study effects on BF%, VO₂max, and VO₂peak. The results showed that the funnel plots for each outcome measure displayed generally good symmetry, indicating that the influence of publication bias or small study effects on the research results was minimal. The funnel plots for body fat percentage and VO₂max showed notable clustering around the center, while the funnel plot for VO₂peak, although containing some scattered points, maintained good overall symmetry, further supporting the stability and reliability of the research results. The analysis results of the outcome measures in this study are credible and were not significantly affected by publication bias or small study effects. The relevant funnel plots are shown in Figure 7.

## 4 Discussion

This study employed systematic review and network meta-analysis to investigate the effects of different exercise interventions on BF%, VO₂max, and VO₂peak in sedentary adults. The findings revealed significant differences in effectiveness among various interventions, specifically showing that AT and combined training (such as RT + ET, AT+ST) were most effective for fat reduction, ST demonstrated superior performance in improving VO₂max, and AT yielded the best results for enhancing VO₂peak.

First, regarding improvements in BF%, this study found that AT demonstrated the highest effectiveness ranking (SUCRA 97.5%) among all exercise modalities, which is consistent with existing research. AT significantly impacts fat breakdown and reduction by increasing energy expenditure and improving basal metabolic rate (23, 24). Combined training methods such as RT + ET and AT+ST also demonstrated good efficacy, possibly due to their dual advantages of combining aerobic exercise and resistance training, which jointly enhance fat metabolism and increase lean body mass (25, 26). Compared to HIIT, AT’s advantage may lie in its broader applicability and higher adherence, especially for the specific population of sedentary individuals (27). Notably, although HIIT has shown good fat reduction effects in some studies, its requirements for individual exercise capacity and tolerance may limit its practical application (28).

For improving VO₂max, this study found ST to be the most significant, markedly higher than other intervention methods. This might be because ST (SUCRA:95.9%) improves cardiopulmonary function by enhancing skeletal muscle strength and improving blood circulation efficiency (29, 30). Additionally, combined training (such as AT+RT, AT+ST) also showed high intervention efficacy, consistent with Gillen et al.’s research on the positive effects of high-intensity and combined training on cardiopulmonary adaptability (31). Although HIIT has shown improvement in VO₂max in some studies, its effect was not significant compared to ST and combined training, possibly because HIIT’s high training intensity has lower adaptability for sedentary individuals with poorer baseline fitness (32).

For VO₂peak improvement, AT demonstrated the highest effectiveness among all interventions (SUCRA: 70.0%), followed by MICT (61.0%) and HIIT (58.9%). This indicates that AT is the most effective exercise modality for enhancing VO₂peak in sedentary adults. This may be related to the fact that improving VO₂peak requires longer intervention cycles and higher intensity training (33). Existing literature indicates that AT has significant long-term benefits for cardiopulmonary function, especially for improving aerobic endurance and metabolic adaptability (34, 35). Additionally, MICT, as a time-saving training mode, shows potential in improving VO₂peak, but more research is needed for verification (36).

In terms of consistency testing, this study did not find significant differences between direct and indirect evidence through methods such as node-splitting and loop inconsistency tests, indicating good consistency of the analysis results (37). Meanwhile, funnel plot analysis showed no obvious publication bias or small study effects for all outcome measures, further supporting the stability and reliability of the research results. This result is consistent with the network meta-analysis methodological studies by Higgins et al. (38) and Salanti et al. (39). The mechanisms by which exercise improves the health status of sedentary populations may involve multiple aspects. First, exercise reduces systemic inflammation levels by promoting fat breakdown and reducing adipose tissue accumulation (40). Long-term aerobic exercise may improve blood supply to adipose tissue by increasing the expression of angiogenins in adipose tissue, reducing hypoxia-induced inflammatory responses. Second, strength training helps improve metabolic disorder states by enhancing skeletal muscle mass and improving insulin sensitivity (41). Additionally, HIIT, as an efficient exercise mode, can significantly improve cardiopulmonary function and metabolic adaptability through short-duration, high-intensity training stimulation (42, 43).

The limitations of this study include the following points: ① Language and publication bias may exist due to the exclusion of non-English studies, potentially skewing results toward populations with greater research output; ② Methodological heterogeneity was observed across studies in measurement techniques (DXA vs. bioelectrical impedance vs. skinfold calipers), intervention protocols, and participant characteristics (I^2^ = 28–41%), which may affect outcome comparability and limit the generalizability of findings; ③ Limited sample sizes for certain interventions, particularly in VO₂peak analysis, and lack of formal inter-rater agreement statistics for risk of bias assessment may affect the stability and reliability of network estimates; ④ Dietary factors were not controlled across studies, while dietary interventions may have synergistic effects with exercise in sedentary populations. Future studies should standardize measurement techniques and incorporate more rigorous methodological approaches to enhance evidence quality.

## 5 Conclusion

This network meta-analysis provides evidence-based exercise prescriptions for sedentary adults based on specific health objectives. AT should be considered the first-line intervention for sedentary adults prioritizing BF% reduction, demonstrating superior effectiveness with the highest SUCRA ranking (97.5%). For individuals seeking to improve VO₂max and cardiovascular capacity, ST emerges as the optimal choice (SUCRA 95.9%), significantly outperforming other modalities. AT also shows promise for enhancing VO₂peak, though evidence remains limited and requires further investigation. Combined training approaches (AT+RT, RT + ET) offer balanced benefits for individuals targeting both fat loss and cardiovascular improvements simultaneously. These findings provide specific guidance for exercise prescription in sedentary populations, supporting individualized intervention strategies based on primary health goals.

## Data Availability

The raw data supporting the conclusions of this article will be made available by the authors, without undue reservation.
