# Supplementary material for: Effects of exercise on body fat percentage and cardiorespiratory fitness in sedentary adults: a systematic review and network meta-analysis
Source: Front Public Health. 2025 Jul 17;13:1624562. doi: 10.3389/fpubh.2025.1624562 (PMC12310449; doi:10.3389/fpubh.2025.1624562)
Supplement: Supplementary file 1 [file Data_Sheet_1.pdf]

Attachment 1

Table 1 Basic Characteristics of Included Studies

Auther	Year	Country	BMI：kg/m2（T/C）		Sedentary criteria	Duration	Group size	Control size	Treatment	Control	Outcome
Kline	2011	United States	≥25		Less than 2 exercise sessions per week	12Weeks	27	16	AT+RT	SE	FP
Per Sjögren	2012	Sweden	≥25		-	6Months	30	43	AT+ST	NN	FP
Aoike	2015	Brazil	31.2±6.1		-	12Weeks	11	8	HICT+RT	HICT+RT	FP
Arad	2015	United States	≥25		Less than 3 times/week, 60 minutes/session	14Weeks	14	14	HIIT	CG	FP、VP
Gillen	2016	Canada	26±6		-	12Weeks	10	6	T1:MICT<br>T2:SIT	CG	FP
Hwang	2016	United States	-		-	8Weeks	T1:15<br>T2:14	14	T1:HIIT<br>T2:MICT	CG	VP、FP
Bartlett	2017	United Kingdom	28.1±6.1	28.1±6.1	-	10Weeks	14	13	HIIT	MICT	VM、FP
Bouaziz	2019	France	28.7 ± 5.3	28.8 ± 5.0	IPAQ: Score < 2	9Weeks	30	30	HIIT	CG	FP
Morita	2019	Japan	18.9﹣23.1	18.7﹣24.0	-	12Weeks	17	12	AT	CG	FP
Nielsen	2019	Denmark	-		-	15Weeks	30	15	AT+ST	CG	VM、FP
Schroeder	2019	United States	T1:32.5±5.9<br>T2:33.1±5.9<br>T3:31.9±5.5	32.4 ±3.7	-	8Weeks	18	17	AT+RT	CG	FP、VM
Michael	2021	United Kingdom	27.80±1.11	20.59±0.59	IPAQ	8Weeks	26	10	HIIT	CG	VM、FP
Bliss	2022	Australia	≥25		-	16Weeks	14	13	AT	CG	FP
Cao	2022	China	-		Sedentary for more than 8 hours per day	7Weeks	35	28	AT+HIIT	CG	FP
De-la-O	2022	Spain	-		-	12Weeks	T1:17<br>T2:16	15	T1:AT+RT<br>T2:HIIT	CG	FP
Garthwaite	2022	Finland	25-40		MVPA < 120 minutes	3Months	33	31	LPA	CG	FP
Callahan	2021	Australia	29±2.6	28.6±3	Not meeting current national PA guidelines for 6 months	6Weeks	T1:12<br>T2:11	12	T1:ET<br>T2:RT	HIIT	FP
Silva	2024	Portugal	26.31 ± 3.51	28.18±4.17	Engaged in sedentary work	16Weeks	18	18	AT+RT	CG	FP、VP
Syamsudin	2023	Indonesia	24.9 ± 4.23	22.9 ± 3.44	IPAQ and PAR-Q questionnaires	2Weeks	7	7	HIIT	CG	VM、FP
Andonian	2024	United States	28-40		-	16Weeks	10	10	AT+RT	NN	FP
Ho	2024	China	-		8 hours per day, 5 days per week	8Weeks	T1:12<br>T2:12	12	T1:HICT<br>T2:AT	CG	FP
Silva	2024	Portugal	26.31 ± 3.51	28.18±4.17	Engaged in sedentary work	16Weeks	18	18	AT+RT	CG	FP、VP
Sun	2024	China	-		-	8Weeks	6	6	T1:MICT<br>T2:HIIT	CG	FP

Cont. Table 3. Basic Characteristics of Included Studies

Auther	Year	国家	BMI：kg/m2（T/C）		Sedentary criteria	Duration	Group size	Control size	Treatment	Control	Outcome
Cakmakci	2011	Turkey	-		-	8Weeks	34	27	AT	CG	FP
Vispute	2011	United States	24.70 ± 3.14	24.47 ± 3.61	-	6Weeks	14	10	MICT	CG	FP
Campbell	2012	United States	28.9±5.5	28.5±4.8	-	12Months	102	100	MICT	CG	FP
Willis	2012	United States	T1:30.5±3.4<br>T2:30.6±3.2	30.5±3.4	Exercise 1－2 times per week	8Months	T1:44<br>T2:38	37	T1:RT<br>T2:AT	RT+AT	FP、VM
Fourie	2013	South Africa	28.32 ± 6.77	29.32±5.44	-	8Weeks	25	25	AT	CG	FP
Baena	2014	Spain	26.9±4.96	26.9±4.96	-	2Months	17	21	AT+RT	NN	FP、VM
Baria	2014	Brazil	30.8 ± 5.1	29.6 ± 1.9	-	12Weeks	10	9	AT	CG	FP、VP
Matsuo	2014	Japan	22.0 ± 1.7	21.7 ± 2.1	No regular exercise in the past year	9Weeks	12	12	HIIT	LIIT	FP
Chiu	2018	China	25.15±3.75	24.85±3.01	-	12Weeks	36	34	ST	NN	FP
Howden	2018	United States	26.2±3.22	25.8±2.96	No history of regular exercise	2Years	33	28	AT	CG	FP
Hunter	2018	United States	31±7	28±7	Moderate physical activity <2 days/week	12Weeks	19	19	AT	CG	FP
Sun	2018	China	22.76±3.56	23.12±2.12	-	5Weeks	10	10	ET	CG	FP
Amaro	2019	Spain	T1:24.7±2.4<br>T2:25.3±2.9	26.4 ± 3.8	MICT <20 minutes, 3 days/week	12Weeks	T1:16<br>T2:16	14	T1:ET+RT<br>T2:HIIT	CG	FP
Krause	2019	Ireland	26.3 ± 2.5	27.2 ± 3.3	-	12Weeks	10	10	RT	CG	FP
Madjd	2019	United Kingdom	31.33±2.55	31.90±2.61	Exercise <3 days/week, <20 minutes/day	24Weeks	32	33	SBP	LPA	FP
Arazi	2020	Iran	29.60 ± 4.49	28.89±3.30	-	8Weeks	10	10	AT	CG	FP
Brennan	2020	United States	37.3 ± 5.4	35.7 ± 4.4	≤1 continuous exercise session/week	6Months	20	20	AT+RT	CG	FP、VP
Briggs	2021	United States	-		Structured PA ≤1 time/week	4Months	11	8	AT+RT	HIIT+RT	FP、VP
Rezaeipour	2021	Iran	-		Exercise frequency: 1－2 times/month or less	2Months	22	22	AT	CG	FP
Nasiri	2022	Iran	-		No regular physical activity or exercise	8Weeks	13	13	AT	CG	FP
Wang	2022	China	23.1 ± 2.6	23.9 ± 1.4	IPAQ	24Weeks	36	12	AT	CG	FP、VM
zd2rgy	2022	United States	T1:33.8±4.1<br>T2:40.3±5.2	30.3 ± 3.7	Exercise <120 minutes/week	4Weeks	T1:HIIT<br>T2:MICT	CG	T1:HIIT<br>T2:MICT	CG	FP、VP
Alemayehu	2023	Ethiopia	T1:27.4±1.8<br>T2:27.53±1.8<br>T3:28.93±1.9	27.81±1.76	-	12Weeks	T1:11<br>T2:11<br>T3:12	12	T1:AT<br>T2:RT<br>T4:AT+RT	CG	FP、VM
Briceño	2023	United States	T1:21.5±3.8<br>T2:22.7±3.5	22.3±3.4	-	8Weeks	T1:10<br>T2:12	12	T1:HIIT<br>T2:MICT	CG	FP

dp727r	2023	United States	29.6±3.73	29.7±4.89	-	6Months	51	53	AT+RT	CG	FP

Cont. Table 3. Basic Characteristics of Included Studies

Auther	Year	国家	BMI：kg/m2（T/C）		Sedentary criteria	Duration	Group size	Control size	Treatment	Control	Outcome
Brandt	2024	United Kingdom	26.9±4.3	28.9±3.8	Sedentary office work	12Months	12	14	RT	CG	FP
Cullen	2024	United States	31.17±4.79	31.26±4.32	Sedentary lifestyle	6Weeks	16	16	RT	CG	FP
Govindasamy	2024	India	T1:29.9±1.3<br>T2:30±1.23	30.18±1.26	No systematic habitual exercise	12Weeks	T1:20<br>T2:20	20	T1:ST<br>T2:RT	CG	FP、VM

2 ROB2

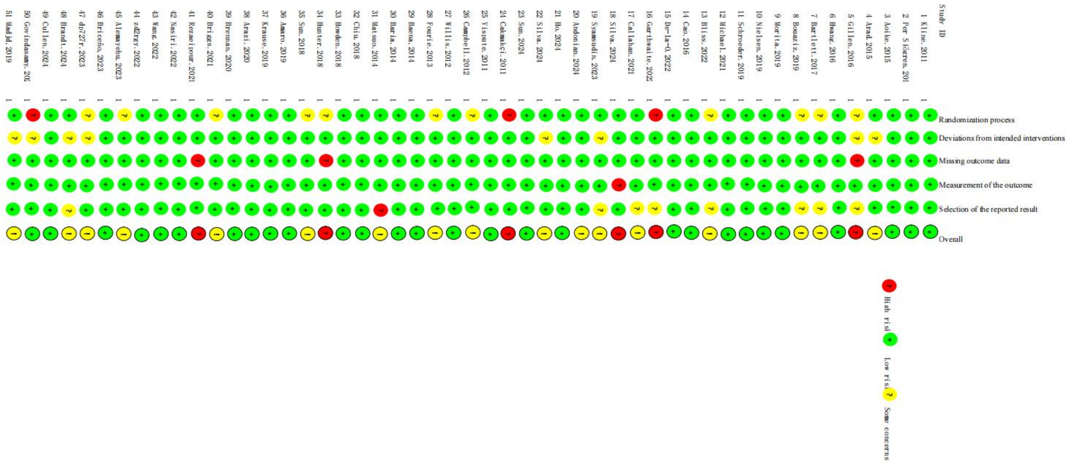

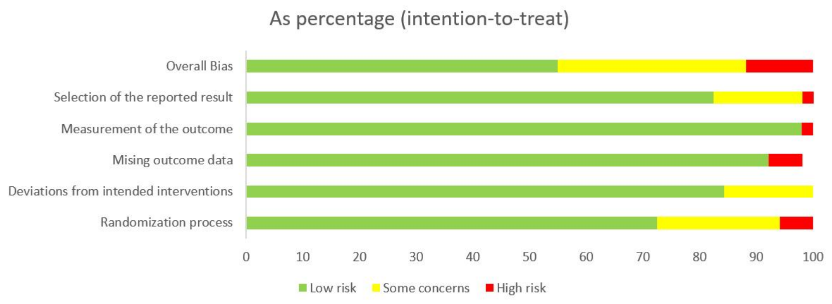

3 Search Query

Table 2 Search Query

Embase	No. Query Results<br>#21 #6 AND #8 AND #12 AND #18 17347<br>#18 'randomized controlled trial'/exp OR random*:ab,ti OR clinic*:ab,ti OR control:ab,ti OR trial:ab,ti 12823977<br>#12 'aged'/exp OR adult* OR 'elderly'/exp OR geriatric:ab,ti OR senior:ab,ti OR frail:ab,ti OR old:ab,ti OR older:ab,ti 13621932<br>#8 'sedentary lifestyle'/exp OR sedentary:ab,ti OR 'physical inactivity':ab,ti OR 'lack of physical activity':ab,ti 77273<br>#6 'exercise'/exp OR 'sport'/exp OR exercise*:ab,ti OR physical:ab,ti OR sport*:ab,ti OR athletic:ab,ti OR train*:ab,ti OR practice:ab,ti 3895608
PuMend	((((((((((Exercise[MeSH Terms]) OR (sports[MeSH Terms])) OR (exercise*[Title/Abstract])) OR (Physical[Title/Abstract])) OR (sport*[Title/Abstract])) OR (Athletic[Title/Abstract])) OR (Train*[Title/Abstract])) OR (Practice[Title/Abstract]) AND (english[Filter])) AND ((((sedentary behavior[MeSH Terms]) OR (Sedentary[Title/Abstract])) OR (physical inactivity[Title/Abstract])) OR (lack of physical activity[Title/Abstract]) AND (english[Filter]))) AND (((((randomized controlled trials as topic[MeSH Terms]) OR (random*[Title/Abstract])) OR (clinic*[Title/Abstract])) OR (control[Title/Abstract])) OR (Trial[Title/Abstract]) AND (english[Filter]))) AND ((((((((Aged[MeSH Terms]) OR (Adult*[MeSH Terms])) OR (Elderly[MeSH Terms])) OR (Geriatric[Title/Abstract])) OR (Senior[Title/Abstract])) OR (frail[Title/Abstract])) OR (old[Title/Abstract])) OR (older[Title/Abstract]) AND (english[Filter]))
Cochrane Library	ID Search Hits<br>#1 MeSH descriptor: [Exercise] explode all trees 39777<br>#2 exercise* or Physical or Athletic 283546<br>#3 MeSH descriptor: [Sports] explode all trees 22575<br>#4 (sport* or Train* or Practice):ti,ab,kw 265918<br>#5 #1 or #2 or #3 or #4 456027<br>#6 MeSH descriptor: [Sedentary Behavior] explode all trees 1914<br>#7 (Sedentary or physical inactivity or lack of physical activity):ti,ab,kw 14754<br>#8 #6 or #7 14754<br>#9 MeSH descriptor: [Randomized Controlled Trials as Topic] explode all trees 56210<br>#10 (random* or clinic* or control or Trial):ti,ab,kw 1810994<br>#11 #9 or #10 1811013<br>#12 MeSH descriptor: [Aged] explode all trees 280953<br>#13 MeSH descriptor: [Adult] explode all trees 627060<br>#14 MeSH descriptor: [Aged] explode all trees 280953<br>#15 (Geriatric or Senior or frail or old or older):ti,ab,kw 172411<br>#16 #12 or #13 or #14 or #15 754720<br>#17 #5 and #8 and #11 and #16 6304

	#	Search Query	Results
Web of Science	1	"exercise* or Physical or sport* or Train* (Topic) OR Athletic or Practice (Topic) AND Sedentary or physical inactivity or lack of physical activity (Topic) AND random* or clinic* or control or Trial (Topic) AND Geriatric or Senior or frail or old or older (Topic) OR Aged or Adult* or Elderly (Topic)	9842804
	2	"exercise* or Physical or sport* or Train* or Athletic or Practice (Topic) AND Sedentary or physical inactivity or lack of physical activity (Topic) AND random* or clinic* or control or Trial (Topic) AND Geriatric or Senior or frail or old or older or Aged or Adult* or Elderly (Topic)	20991
	3	"exercise* or Physical or sport* or Train* or Athletic or Practice (Topic) AND Sedentary or physical inactivity or lack of physical activity (Topic) AND random* or clinic* or control or Trial (Topic) AND Geriatric or Senior or frail or old or older or Aged or Adult* or Elderly (Topic) and 2025 or 2024 or 2023 or 2022 or 2021 or 2020 or 2019 or 2018 or 2017 or 2016 or 2015 or 2014 or 2013 or 2012 or 2010 or 2011 or 2009 (Publication Years)	17300
	4	"exercise* or Physical or sport* or Train* or Athletic or Practice (Topic) AND Sedentary or physical inactivity or lack of physical activity (Topic) AND random* or clinic* or control or Trial (Topic) AND Geriatric or Senior or frail or old or older or Aged or Adult* or Elderly (Topic) and 2025 or 2024 or 2023 or 2022 or 2021 or 2020 or 2019 or 2018 or 2017 or 2016 or 2015 or 2014 or 2013 or 2012 or 2010 or 2011 or 2009 (Publication Years) and English (Languages)	16939

Table 3 Body Fat Percentage (BF%)

ST	SIT	SE	SBP	RT	NN	MICT	LPA	LIIT	HIIT	HICT	ET	CT	CG	AT_ST	AT_RT	AT_HIIT	AT_ET	AT	AEX_RT
ST	1.58 (-1.44, 4.59)	3.08 (-1.39, 7.54)	0.72 (-1.60, 3.03)	1.81 (-1.92, 5.54)	2.44 (0.53, 4.35)	-1.62 (-7.93, 4.68)	1.43 (0.04, 2.82)	2.16 (-1.05, 5.38)	2.68 (-1.28, 6.63)	2.78 (-3.71, 9.26)	1.91 (0.06, 3.76)	-0.20 (-6.42, 6.02)	3.04 (-0.80, 6.89)	0.99 (-0.39, 2.37)	1.94 (-3.73, 7.60)	3.14 (-0.20, 6.48)	1.03 (-1.57, 3.63)	2.68 (1.58, 3.77)	-0.32 (-7.68, 7.03)
-1.58 (-4.59, 1.44)	SIT	1.50 (-3.66, 6.66)	-0.86 (-4.33, 2.61)	0.23 (-4.31, 4.78)	0.86 (-2.36, 4.09)	-3.20 (-10.01, 3.61)	-0.14 (-3.15, 2.87)	0.59 (-3.62, 4.80)	1.10 (-3.63, 5.83)	1.20 (-5.79, 8.19)	0.34 (-2.85, 3.53)	-1.78 (-8.68, 5.13)	1.47 (-3.22, 6.15)	-0.59 (-3.59, 2.42)	0.36 (-5.86, 6.58)	1.56 (-2.67, 5.80)	-0.55 (-4.26, 3.17)	1.10 (-1.71, 3.91)	-1.90 (-9.70, 5.90)
-3.08 (-7.54, 1.39)	-1.50 (-6.66, 3.66)	SE	-2.36 (-7.14, 2.42)	-1.27 (-6.88, 4.34)	-0.64 (-5.24, 3.97)	-4.70 (-12.26, 2.86)	-1.64 (-6.10, 2.82)	-0.91 (-6.25, 4.43)	-0.40 (-6.16, 5.36)	-0.30 (-8.02, 7.42)	-1.16 (-5.74, 3.42)	-3.28 (-10.93, 4.38)	-0.03 (-5.75, 5.69)	-2.09 (-6.54, 2.37)	-1.14 (-8.18, 5.90)	0.06 (-5.29, 5.42)	-2.05 (-7.01, 2.91)	-0.40 (-4.73, 3.93)	-3.40 (-11.86, 5.06)
-0.72 (-3.03, 1.60)	0.86 (-2.61, 4.33)	2.36 (-2.42, 7.14)	SBP	1.09 (-3.02, 5.20)	1.72 (-0.86, 4.30)	-2.34 (-8.87, 4.19)	0.72 (-1.59, 3.02)	1.45 (-2.29, 5.18)	1.96 (-2.36, 6.27)	2.06 (-4.65, 8.77)	1.20 (-1.34, 3.74)	-0.92 (-7.55, 5.71)	2.33 (-1.94, 6.59)	0.27 (-2.03, 2.57)	1.22 (-4.70, 7.13)	2.42 (-1.34, 6.19)	0.31 (-2.86, 3.48)	1.96 (-0.08, 3.99)	-1.04 (-8.59, 6.51)
6.00 (0.36, 11.65)	7.58 (1.37, 13.79)	9.08 (2.05, 16.11)	6.72 (0.82, 12.62)	7.81 (1.22, 14.40)	8.44 (2.69, 14.20)	4.38 (-3.94, 12.70)	7.44 (1.80, 13.08)	8.17 (1.81, 14.53)	8.68 (1.96, 15.40)	8.78 (0.32, 17.24)	7.92 (2.18, 13.66)	5.80 (-2.59, 14.20)	9.05 (2.36, 15.74)	6.99 (1.36, 12.63)	7.94 (0.10, 15.78)	9.14 (2.77, 15.52)	7.03 (0.99, 13.08)	8.68 (3.14, 14.22)	5.68 (-3.46, 14.82)
-1.81 (-5.54, 1.92)	-0.23 (-4.78, 4.31)	1.27 (-4.34, 6.88)	-1.09 (-5.20, 3.02)	RT	0.63 (-3.21, 4.47)	-3.43 (-10.59, 3.73)	-0.37 (-4.08, 3.33)	0.36 (-4.39, 5.11)	0.87 (-4.35, 6.09)	0.97 (-6.36, 8.29)	0.11 (-3.43, 3.64)	-2.01 (-9.26, 5.24)	1.24 (-3.93, 6.40)	-0.82 (-4.54, 2.90)	0.13 (-6.47, 6.73)	1.33 (-3.38, 6.05)	-0.78 (-5.09, 3.54)	0.87 (-2.70, 4.44)	-2.13 (-10.23, 5.97)
-2.44 (-4.35, -0.53)	-0.86 (-4.09, 2.36)	0.64 (-3.97, 5.24)	-1.72 (-4.30, 0.86)	-0.63 (-4.47, 3.21)	NN	-4.06 (-10.47, 2.34)	-1.01 (-2.91, 0.89)	-0.27 (-3.78, 3.23)	0.24 (-3.89, 4.36)	0.34 (-6.25, 6.92)	-0.53 (-2.50, 1.45)	-2.64 (-9.14, 3.86)	0.60 (-3.46, 4.67)	-1.45 (-3.35, 0.45)	-0.50 (-6.28, 5.27)	0.70 (-2.04, 3.44)	-1.41 (-4.31, 1.49)	0.24 (-1.35, 1.82)	-2.76 (-10.21, 4.68)
1.62 (-4.68, 7.93)	3.20 (-3.61, 10.01)	4.70 (-2.86, 12.26)	2.34 (-4.19, 8.87)	3.43 (-3.73, 10.59)	4.06 (-2.34, 10.47)	MICT	3.06 (-3.24, 9.36)	3.79 (-3.16, 10.74)	4.30 (-2.98, 11.58)	4.40 (-4.51, 13.31)	3.54 (-2.85, 9.93)	1.42 (-7.43, 10.28)	4.67 (-2.58, 11.92)	2.61 (-3.68, 8.91)	3.56 (-4.77, 11.89)	4.76 (-2.20, 11.73)	2.65 (-4.01, 9.32)	4.30 (-1.90, 10.50)	1.30 (-8.26, 10.86)
-1.43 (-2.82, -0.04)	0.14 (-2.87, 3.15)	1.64 (-2.82, 6.10)	-0.72 (-3.02, 1.59)	0.37 (-3.33, 4.08)	1.01 (-0.89, 2.91)	-3.06 (-9.36, 3.24)	LPA	0.73 (-2.56, 4.03)	1.24 (-2.71, 5.20)	1.34 (-5.14, 7.83)	0.48 (-1.29, 2.25)	-1.63 (-8.00, 4.74)	1.61 (-2.10, 5.32)	-0.44 (-1.92, 1.03)	0.50 (-5.16, 6.16)	1.71 (-1.63, 5.04)	-0.40 (-3.05, 2.24)	1.24 (0.16, 2.32)	-1.76 (-9.11, 5.59)
-2.16 (-5.38, 1.05)	-0.59 (-4.80, 3.62)	0.91 (-4.43, 6.25)	-1.45 (-5.18, 2.29)	-0.36 (-5.11, 4.39)	0.27 (-3.23, 3.78)	-3.79 (-10.74, 3.16)	-0.73 (-4.03, 2.56)	LIIT	0.51 (-4.42, 5.44)	0.61 (-6.51, 7.73)	-0.25 (-3.73, 3.23)	-2.36 (-9.36, 4.63)	0.88 (-3.99, 5.75)	-1.18 (-4.46, 2.11)	-0.23 (-6.61, 6.15)	0.97 (-3.47, 5.42)	-1.14 (-3.53, 1.26)	0.51 (-2.62, 3.64)	-2.49 (-10.41, 5.43)
-2.68 (-6.63, 1.28)	-1.10 (-5.83, 3.63)	0.40 (-5.36, 6.16)	-1.96 (-6.27, 2.36)	-0.87 (-6.09, 4.35)	-0.24 (-4.36, 3.89)	-4.30 (-11.58, 2.98)	-1.24 (-5.20, 2.71)	-0.51 (-5.44, 4.42)	HIIT	0.10 (-7.34, 7.54)	-0.76 (-4.86, 3.34)	-2.88 (-10.24, 4.49)	0.37 (-4.97, 5.71)	-1.69 (-5.64, 2.26)	-0.74 (-7.47, 5.99)	0.46 (-4.48, 5.41)	-1.65 (-6.16, 2.87)	-0.00 (-3.81, 3.81)	-3.00 (-11.21, 5.21)
-2.78 (-9.26, 3.71)	-1.20 (-8.19, 5.79)	0.30 (-7.42, 8.02)	-2.06 (-8.77, 4.65)	-0.97 (-8.29, 6.36)	-0.34 (-6.92, 6.25)	-4.40 (-13.31, 4.51)	-1.34 (-7.83, 5.14)	-0.61 (-7.73, 6.51)	-0.10 (-7.54, 7.34)	HICT	-0.86 (-7.43, 5.71)	-2.98 (-11.96, 6.01)	0.27 (-7.14, 7.68)	-1.79 (-8.27, 4.70)	-0.84 (-9.31, 7.63)	0.36 (-6.77, 7.50)	-1.75 (-8.59, 5.09)	-0.10 (-6.49, 6.29)	-3.10 (-12.78, 6.58)
-1.91 (-3.76, -0.06)	-0.34 (-3.53, 2.85)	1.16 (-3.42, 5.74)	-1.20 (-3.74, 1.34)	-0.11 (-3.64, 3.43)	0.53 (-1.45, 2.50)	-3.54 (-9.93, 2.85)	-0.48 (-2.25, 1.29)	0.25 (-3.23, 3.73)	0.76 (-3.34, 4.86)	0.86 (-5.71, 7.43)	ET	-2.11 (-8.60, 4.37)	1.13 (-2.89, 5.15)	-0.93 (-2.77, 0.92)	0.02 (-5.74, 5.78)	1.23 (-2.15, 4.60)	-0.89 (-3.74, 1.97)	0.76 (-0.76, 2.28)	-2.24 (-9.67, 5.19)
0.20 (-6.02, 6.42)	1.78 (-5.13, 8.68)	3.28 (-4.38, 10.93)	0.92 (-5.71, 7.55)	2.01 (-5.24, 9.26)	2.64 (-3.86, 9.14)	-1.42 (-10.28, 7.43)	1.63 (-4.74, 8.00)	2.36 (-4.63, 9.36)	2.88 (-4.49, 10.24)	2.98 (-6.01, 11.96)	2.11 (-4.37, 8.60)	CT	3.24 (-4.07, 10.56)	1.19 (-5.18, 7.56)	2.14 (-6.27, 10.54)	3.34 (-3.72, 10.40)	1.23 (-5.51, 7.97)	2.88 (-3.44, 9.19)	-0.12 (-9.75, 9.50)
-3.04 (-6.89, 0.80)	-1.47 (-6.15, 3.22)	0.03 (-5.69, 5.75)	-2.33 (-6.59, 1.94)	-1.24 (-6.40, 3.93)	-0.60 (-4.67, 3.46)	-4.67 (-11.92, 2.58)	-1.61 (-5.32, 2.10)	-0.88 (-5.75, 3.99)	-0.37 (-5.71, 4.97)	-0.27 (-7.68, 7.14)	-1.13 (-5.15, 2.89)	-3.24 (-10.56, 4.07)	CG	-2.06 (-5.82, 1.71)	-1.11 (-7.81, 5.59)	0.10 (-4.80, 4.99)	-2.02 (-6.47, 2.44)	-0.37 (-4.12, 3.38)	-3.37 (-11.55, 4.81)
-0.99 (-2.37, 0.39)	0.59 (-2.42, 3.59)	2.09 (-2.37, 6.54)	-0.27 (-2.57, 2.03)	0.82 (-2.90, 4.54)	1.45 (-0.45, 3.35)	-2.61 (-8.91, 3.68)	0.44 (-1.03, 1.92)	1.18 (-2.11, 4.46)	1.69 (-2.26, 5.64)	1.79 (-4.70, 8.27)	0.93 (-0.92, 2.77)	-1.19 (-7.56, 5.18)	2.06 (-1.71, 5.82)	AT+ST	0.95 (-4.71, 6.60)	2.15 (-1.18, 5.49)	0.04 (-2.59, 2.68)	1.69 (0.63, 2.74)	-1.31 (-8.66, 6.03)
-1.94 (-7.60, 3.73)	-0.36 (-6.58, 5.86)	1.14 (-5.90, 8.18)	-1.22 (-7.13, 4.70)	-0.13 (-6.73, 6.47)	0.50 (-5.27, 6.28)	-3.56 (-11.89, 4.77)	-0.50 (-6.16, 5.16)	0.23 (-6.15, 6.61)	0.74 (-5.99, 7.47)	0.84 (-7.63, 9.31)	-0.02 (-5.78, 5.74)	-2.14 (-10.54, 6.27)	1.11 (-5.59, 7.81)	-0.95 (-6.60, 4.71)	AT+RT	1.20 (-5.19, 7.60)	-0.91 (-6.97, 5.15)	0.74 (-4.81, 6.29)	-2.26 (-11.41, 6.89)
-3.14 (-6.48, 0.20)	-1.56 (-5.80, 2.67)	-0.06 (-5.42, 5.29)	-2.42 (-6.19, 1.34)	-1.33 (-6.05, 3.38)	-0.70 (-3.44, 2.04)	-4.76 (-11.73, 2.20)	-1.71 (-5.04, 1.63)	-0.97 (-5.42, 3.47)	-0.46 (-5.41, 4.48)	-0.36 (-7.50, 6.77)	-1.23 (-4.60, 2.15)	-3.34 (-10.40, 3.72)	-0.10 (-4.99, 4.80)	-2.15 (-5.49, 1.18)	-1.20 (-7.60, 5.19)	AT+HIIT	-2.11 (-6.10, 1.88)	-0.46 (-3.63, 2.70)	-3.46 (-11.39, 4.46)
-1.03 (-3.63, 1.57)	0.55 (-3.17, 4.26)	2.05 (-2.91, 7.01)	-0.31 (-3.48, 2.86)	0.78 (-3.54, 5.09)	1.41 (-1.49, 4.31)	-2.65 (-9.32, 4.01)	0.40 (-2.24, 3.05)	1.14 (-1.26, 3.53)	1.65 (-2.87, 6.16)	1.75 (-5.09, 8.59)	0.89 (-1.97, 3.74)	-1.23 (-7.97, 5.51)	2.02 (-2.44, 6.47)	-0.04 (-2.68, 2.59)	0.91 (-5.15, 6.97)	2.11 (-1.88, 6.10)	AT+ET	1.65 (-0.78, 4.07)	-1.35 (-9.02, 6.31)
-2.68 (-3.77, -1.58)	-1.10 (-3.91, 1.71)	0.40 (-3.93, 4.73)	-1.96 (-3.99, 0.08)	-0.87 (-4.44, 2.70)	-0.24 (-1.82, 1.35)	-4.30 (-10.50, 1.90)	-1.24 (-2.32, -0.16)	-0.51 (-3.64, 2.62)	0.00 (-3.81, 3.81)	0.10 (-6.29, 6.49)	-0.76 (-2.28, 0.76)	-2.88 (-9.19, 3.44)	0.37 (-3.38, 4.12)	-1.69 (-2.74, -0.63)	-0.74 (-6.29, 4.81)	0.46 (-2.70, 3.63)	-1.65 (-4.07, 0.78)	AT	-3.00 (-10.27, 4.27)
0.32 (-7.03, 7.68)	1.90 (-5.90, 9.70)	3.40 (-5.06, 11.86)	1.04 (-6.51, 8.59)	2.13 (-5.97, 10.23)	2.76 (-4.68, 10.21)	-1.30 (-10.86, 8.26)	1.76 (-5.59, 9.11)	2.49 (-5.43, 10.41)	3.00 (-5.21, 11.21)	3.10 (-6.58, 12.78)	2.24 (-5.19, 9.67)	0.12 (-9.50, 9.75)	3.37 (-4.81, 11.55)	1.31 (-6.03, 8.66)	2.26 (-6.89, 11.41)	3.46 (-4.46, 11.39)	1.35 (-6.31, 9.02)	3.00 (-4.27, 10.27)	AEX+RT

Table 4 Maximum Oxygen Uptake (VO_2 max)

ST_	RT_	MICT_	HIIT_	ET_	CG_	AT_ST_	AT_RT_	AT_
ST	-0.30 (-6.86, 6.26)	1.22 (-5.88, 8.32)	2.51 (-2.38, 7.39)	3.19 (-1.73, 8.10)	4.92 (1.45, 8.39)	6.02 (2.89, 9.15)	7.37 (3.96, 10.77)	11.29 (5.00, 17.58)
0.30 (-6.26, 6.86)	RT	1.52 (-8.15, 11.19)	2.81 (-5.37, 10.99)	3.49 (-4.71, 11.68)	5.22 (-2.20, 12.64)	6.32 (-0.95, 13.59)	7.67 (0.27, 15.06)	11.59 (2.50, 20.68)
-1.22 (-8.32, 5.88)	-1.52 (-11.19, 8.15)	MICT	1.29 (-7.33, 9.91)	1.97 (-6.67, 10.60)	3.70 (-4.20, 11.61)	4.80 (-2.96, 12.56)	6.15 (-1.73, 14.02)	10.07 (0.58, 19.56)
-2.51 (-7.39, 2.38)	-2.81 (-10.99, 5.37)	-1.29 (-9.91, 7.33)	HIIT	0.68 (-6.25, 7.60)	2.41 (-3.60, 8.42)	3.51 (-2.29, 9.31)	4.86 (-1.10, 10.81)	8.78 (0.82, 16.75)
-2.21 (-11.67, 7.25)	-2.51 (-14.02, 9.00)	-0.99 (-12.82, 10.84)	0.30 (-7.80, 8.40)	0.98 (-9.68, 11.64)	2.71 (-7.37, 12.80)	3.81 (-6.15, 13.78)	5.16 (-4.90, 15.21)	9.08 (-2.28, 20.44)
-3.19 (-8.10, 1.73)	-3.49 (-11.68, 4.71)	-1.97 (-10.60, 6.67)	-0.68 (-7.60, 6.25)	ET	1.73 (-4.29, 7.75)	2.83 (-2.99, 8.66)	4.18 (-1.80, 10.16)	8.10 (0.12, 16.08)
-4.92 (-8.39, -1.45)	-5.22 (-12.64, 2.20)	-3.70 (-11.61, 4.20)	-2.41 (-8.42, 3.60)	-1.73 (-7.75, 4.29)	CG	1.10 (-3.41, 5.61)	2.44 (-1.68, 6.57)	6.37 (-0.82, 13.55)
-6.02 (-9.15, -2.89)	-6.32 (-13.59, 0.95)	-4.80 (-12.56, 2.96)	-3.51 (-9.31, 2.29)	-2.83 (-8.66, 2.99)	-1.10 (-5.61, 3.41)	AT+ST	1.34 (-2.65, 5.34)	5.27 (-1.76, 12.29)
-7.37 (-10.77, -3.96)	-7.67 (-15.06, -0.27)	-6.15 (-14.02, 1.73)	-4.86 (-10.81, 1.10)	-4.18 (-10.16, 1.80)	-2.44 (-6.57, 1.68)	-1.34 (-5.34, 2.65)	AT+RT	3.92 (-3.23, 11.08)
-11.29 (-17.58, -5.00)	-11.59 (-20.68, -2.50)	-10.07 (-19.56, -0.58)	-8.78 (-16.75, -0.82)	-8.10 (-16.08, -0.12)	-6.37 (-13.55, 0.82)	-5.27 (-12.29, 1.76)	-3.92 (-11.08, 3.23)	AT

Table 5 Peak Oxygen Uptake (VO_2 peak)

SE	1.91 (-4.73, 8.54)	1.98 (-1.90, 5.87)	0.41 (-7.14, 7.95)	-0.09 (-2.74, 2.57)	-0.39 (-2.63, 1.85)
-1.91 (-8.54, 4.73)	MICT	0.08 (-6.93, 7.09)	-1.50 (-5.10, 2.10)	-1.99 (-8.80, 4.81)	-2.30 (-8.55, 3.95)
-1.98 (-5.87, 1.90)	-0.08 (-7.09, 6.93)	HIIT	-1.58 (-9.45, 6.30)	-2.07 (-6.23, 2.09)	-2.38 (-5.56, 0.81)
-0.41 (-7.95, 7.14)	1.50 (-2.10, 5.10)	1.58 (-6.30, 9.45)	CG	-0.49 (-8.19, 7.20)	-0.80 (-8.01, 6.41)
0.09 (-2.57, 2.74)	1.99 (-4.81, 8.80)	2.07 (-2.09, 6.23)	0.49 (-7.20, 8.19)	AT+RT	-0.31 (-3.00, 2.39)
0.39 (-1.85, 2.63)	2.30 (-3.95, 8.55)	2.38 (-0.81, 5.56)	0.80 (-6.41, 8.01)	0.31 (-2.39, 3.00)	AT

Supplementary Table 6. Network Meta-Analysis Consistency and Heterogeneity Assessment Results

Outcome Measure	I^2 (%)	τ^2	Heterogeneity Level	Design-by-Treatment Inconsistency	Cochran's Q Test	Model Used
Body Fat Percentage	32%	0.08	Low to moderate	P = 0.87 (consistent)	P = 0.24	Random-effects model
Maximal Oxygen Uptake	28%	0.06	Low	P = 0.62 (consistent)	P = 0.31	Random-effects model
Peak Oxygen Uptake	41%	0.12	Moderate	P = 0.70 (consistent)	P = 0.18	Random-effects model

4 Figure

Body Fat Percentage (BF%) Maximum Oxygen Uptake (VO_2max) Peak Oxygen Uptake (VO_2peak)

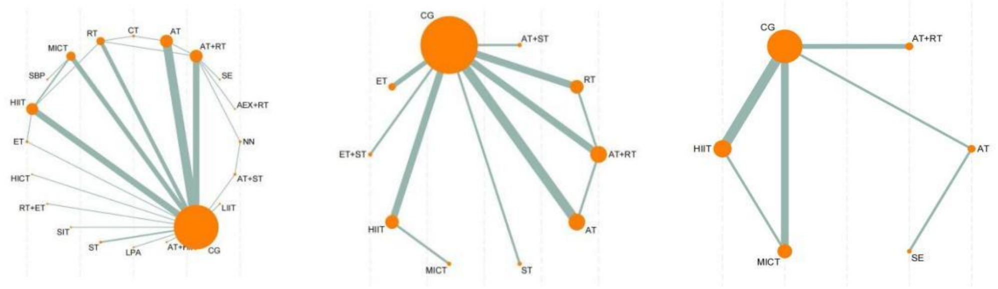

Figure 3. Network Evidence Plot of Different Exercise Interventions on Body Fat Percentage, Maximum

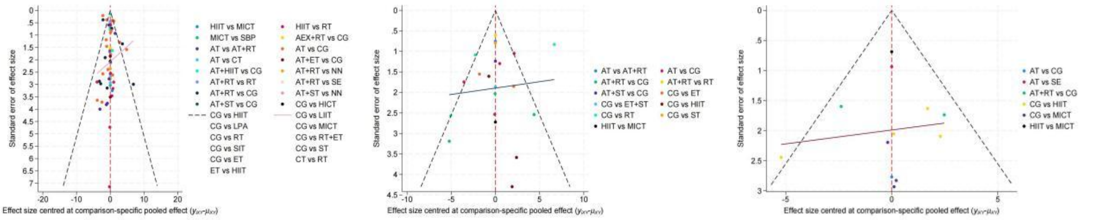

Figure 6. Funnel Plots for the Effectiveness Rankings of Exercise Interventions on Body Fat Percentage, Maximal Oxygen Uptake (VO_2max), and Peak Oxygen Uptake (VO_2peak)

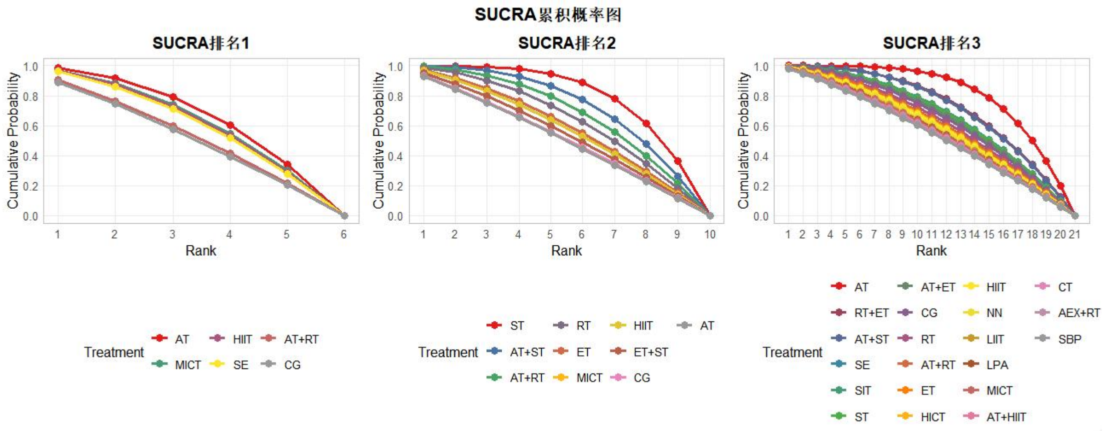

Figure 6. Cumulative Probability Ranking Diagram of Various Exercise Interventions on Peak Oxygen Uptake, Maximal Oxygen Uptake, and Body Fat Percentage

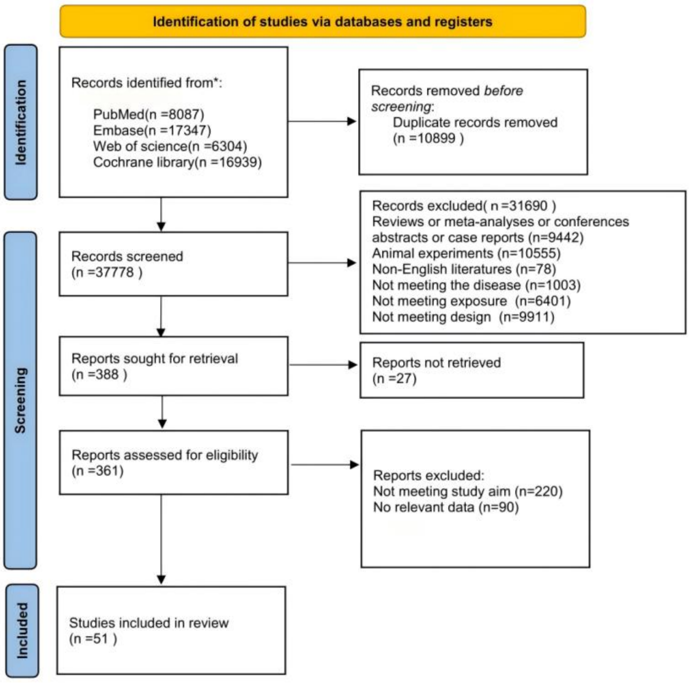

Figure 1 PRISMA flow diagram

5 Table

Table 1 Inclusion and Exclusion Criteria

Category	Specific Criteria
Inclusion Criteria	Randomized controlled trials (RCTs), limited to Chinese and English languages. Sedentary adults, with sedentary behavior defined as self-reported or assessed by physical activity questionnaires, with average daily sedentary time ≥6 hours, or not engaging in regular physical activity (moderate-intensity activity <120 minutes per week). Control groups consisting of blank control (no intervention), health education, or exercise modalities different from the intervention group. Assessment of at least one primary outcome measure before and after intervention: body fat percentage (%), maximum oxygen uptake (VO_2max), or peak oxygen uptake (VO_2peak).

Exclusion Criteria	Patients with diabetes. Theses, conference abstracts, registration protocols, animal experimental studies, and literature for which full texts were unavailable. Studies from which valid data could not be extracted, and attempts to contact the authors were unsuccessful. Duplicate literature content or repeatedly published research data. Studies where participants were not defined as sedentary adults, or where sedentary behavior was not clearly reported.
